# Supplementary material for: Novel Carboxylation Method for Polyetheretherketone (PEEK) Surface Modification Using Friedel–Crafts Acylation
Source: Int J Mol Sci. 2023 Oct 27;24(21):15651. doi: 10.3390/ijms242115651 (PMC10650194; doi:10.3390/ijms242115651)
Supplement: Supplementary file 1 [file ijms-24-15651-s001.zip › ijms-2652036-supplementary.pdf]

International Journal of *Molecular Sciences*
MDPI

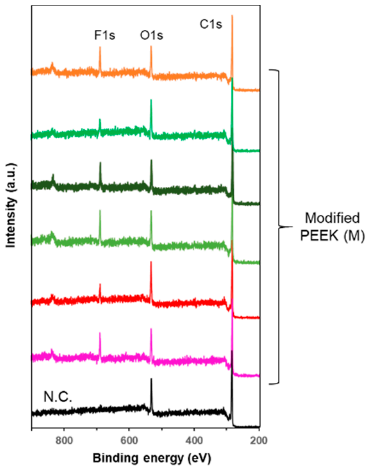

Figure S1. XPS spectral results of N.C. and all M group samples. Clear F1s signals are observed in all M group samples.

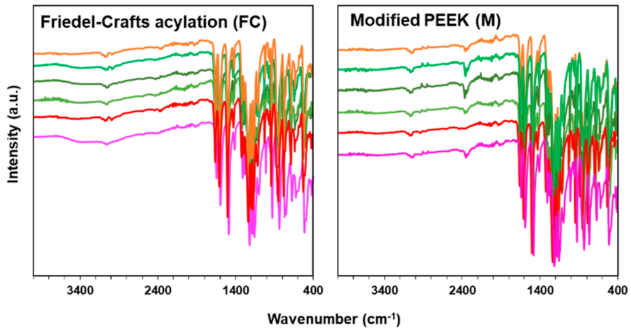

Figure S2. FT-IR results for all FC and M group samples. The same color indicates that the samples correspond between FC and M, respectively. The absorption bands appeared in the wavenumber region at approximately 3300–3500 cm^{-1} , whtch are observed in the FC group, while they disappeared or weakened in all M group samples.
